# A scoping review of abdominal wall functional assessment in patients with hernias

**DOI:** 10.1007/s10029-025-03517-2

**Published:** 2025-11-24

**Authors:** Haiye Shen, Dominic Farris, David L. Sanders, Helen Dawes, Sarah E. Lamb, John M. Findlay

**Affiliations:** 1 Department of Clinical and Biomedical Sciences, Faculty of Health and Life Sciences, University of Exeter, Exeter, Exeter, EX1 2LU UK; 2 NIHR Exeter Biomedical Research Centre, University of Exeter Medical School. St Luke’s Campus, Exeter, EX1 2LU UK; 3 Department of Public Health and Sport Sciences, Faculty of Health and Life Sciences, University of Exeter, Exeter, EX1 2LU UK; 4 Academic Department of Abdominal Wall Surgery, North Devon District Hospital, Royal Devon University Healthcare NHS Foundation Trust, Barnstaple, EX31 4JB UK; 5 College of Medicine and Health, University of Exeter Medical School, St Luke’s Campus, Exeter, EX1 2LU UK

**Keywords:** Hernia, Incisional hernia, Ventral hernia, Abdominal wall function, Strength

## Abstract

**Background:**

Impairments in abdominal wall function are common in patients with hernias, with negative effects on their quality of life, often requiring surgical repair. Despite advances in repair techniques, there is no consistent standard for assessing abdominal wall function throughout the peri-operative period, limiting comparability between treatments. The aim of this scoping review was to identify and appraise all the available assessments of abdominal wall function reported in the literature, with a focus on the assessment methods and tools used.

**Objectives:**

To identify and review the assessment methods and tools used to measure abdominal wall function.

**Eligibility criteria:**

All studies investigating abdominal wall function through any type of assessment were included. There was no restriction on the year of publication.

**Source of evidence:**

A literature search was performed of the PubMed, EMBASE, and Cochrane Central Register of Controlled Trials databases in June 2025.

**Charting methods:**

Data was extracted independently by two reviewers. Extracted data included assessment tools and functional domains.

**Results:**

18 studies were included, where the majority (88.9%) of them focused on the assessment of abdominal muscle strength. Other aspects of abdominal wall function, such as trunk mobility, core stability, and postural control, were also reported but less frequently. There was considerable heterogeneity in how and when abdominal wall function was assessed and defined.

**Conclusion:**

This scoping review identified substantial variation in assessments of abdominal wall function. The evaluation of abdominal muscle strength remains dominant, but there is inconsistency regarding the tools and tests used, including isokinetic or isometric dynamometers, surface electromyography (sEMG), and clinical tests such as double leg lowering and trunk raising tests, assessed at different postoperative timepoints. No comprehensive or standardized method currently exists to evaluate abdominal wall function across domains and timepoints, underscoring a critical gap in clinical and research practice. Future research needs to develop or modify existing assessment methods to reflect abdominal wall function more holistically.

## Introduction

 The normal function of the abdominal wall is achieved through the coordinated interactions of the muscles, tendons, fascia, spine, and pelvic floor. It regulates intra-abdominal pressure (IAP), provides support for the contents of the abdominal cavity, and promotes core stability and dynamic control, which are fundamental to body movements and daily activities [1].

Impairments in global abdominal wall function (AWF) are common in patients with primary ventral and incisional hernias, with negative effects on their quality of life (QoL) [2]. The weakness or defect in their muscles or fascia affects the structural integrity of the abdominal wall, normal tissue compliance, muscle function, orientation and coordination which often requires surgical repair [3]. Despite advances in repair techniques aimed at improving surgical outcomes [4], there is no consistent standard for assessing AWF pre- and post-operation, which is a key determinant of ‘success’ of surgery. Measurements vary significantly in terms of the methods and tools used. ‘Function’ as a whole often forms a part of patient-reported outcome measures (PROMS), however, in contrast to other components (such as pain, activity and mood) lacks an accepted assessment method.

The most recent systematic review in this field focused on abdominal muscle strength only, where other domains of AWF were not investigated [5]. As there is no consensus on the definition and domains of AWF currently, other domains may include, but are not limited to, postural control, trunk mobility, and core stability. Therefore, this study aims to identify and appraise all the available assessments of AWF, rather than limiting the scope solely to abdominal muscle strength. The findings of this review may contribute to the development of a comprehensive assessment of AWF, which could be applied to clinical practice, and in research to provide a novel outcome measure to develop and compare interventions before, during and after surgery.

## Methods

This review was conducted based on a pre-established protocol developed following the Preferred Reporting Items for Systematic Reviews and Meta-Analyses (PRISMA) guidelines [6]. The protocol for this review has been registered in PROSPERO (Registration ID: CRD420251059333).

### Eligibility criteria

All studies investigating AWF through any type of assessment were included if they contained original data on AWF assessment. There were no restrictions on research design, while systematic reviews, case reports, and animal studies were excluded. Studies that reported only non-functional outcomes (e.g., imaging without functional correlation), or those that assessed unrelated body regions (e.g., lower limb strength only) were excluded.

### Search strategy

A literature search was conducted in data bases including PubMed, EMBASE and Cochrane Central Register of Controlled Trials (CENTRAL) applying the following five groups of search terms: (hernia or abdominal wall reconstruction), (abdomen or abdominal or core or abdomini*), (muscle or muscular), (function OR strength OR activity OR performance OR movement OR contraction OR stability), (test OR assessment OR evaluation OR measurement). They were combined with the Boolean Operators ‘AND’ to narrow the search results. There was no restriction on the year of publication, as this review aims to identify and capture all assessments targeted at AWF that have been used in practice. Bibliographies of retrieved articles will be searched, along with Clinical Trials.gov to identify any further potential studies. The online version of Rayyan (Rayyan, Cambridge, MA, USA) was used to remove duplicate records, and allow manual screening of the remaining results [7]. All references were managed using EndNote 21(Clarivate Analytics).

### Selection of studies

All retrieved results were initially screened by title and abstract, and reviewed independently by two authors (HS and JMF). The relevant studies were further reviewed in full text. Any differences of opinion were resolved through discussion among the reviewers.

### Data extraction

Data collection was carried out independently by two investigators (HS and JMF). The primary endpoints were all assessment methods and tools reported by the included studies. In addition, data were collected regarding research design, participant characteristics, and targeted domain of AWF (e.g., strength, mobility). Where available, data were also extracted on the application of patient-reported outcomes, such as QoL. Furthermore, data regarding the reliability and validity of the assessments were extracted where reported.

### Data analysis

Due to the heterogeneity of research designs, surgical techniques, and assessment methods, a narrative synthesis without meta-analysis (SWIM) was conducted to describe and compare the assessments used to measure AWF in the included studies. Descriptive statistics were used to summarize study and population characteristics. Assessment tools were grouped according to the type of functional domain assessed. Where available, measurements were mapped to components of the COnsensus-based Standards for the selection of health Measurement Instruments (COSMIN) framework, including aspects of validity, reliability, and feasibility in clinical settings. As the aim of this review was to map and summarize existing evidence on AWF assessment methods and tools rather than to evaluate study quality, and in accordance with the PRISMA-ScR guidelines [8], no formal quality or risk of bias assessment was conducted.

## Results

**Fig. 1:**
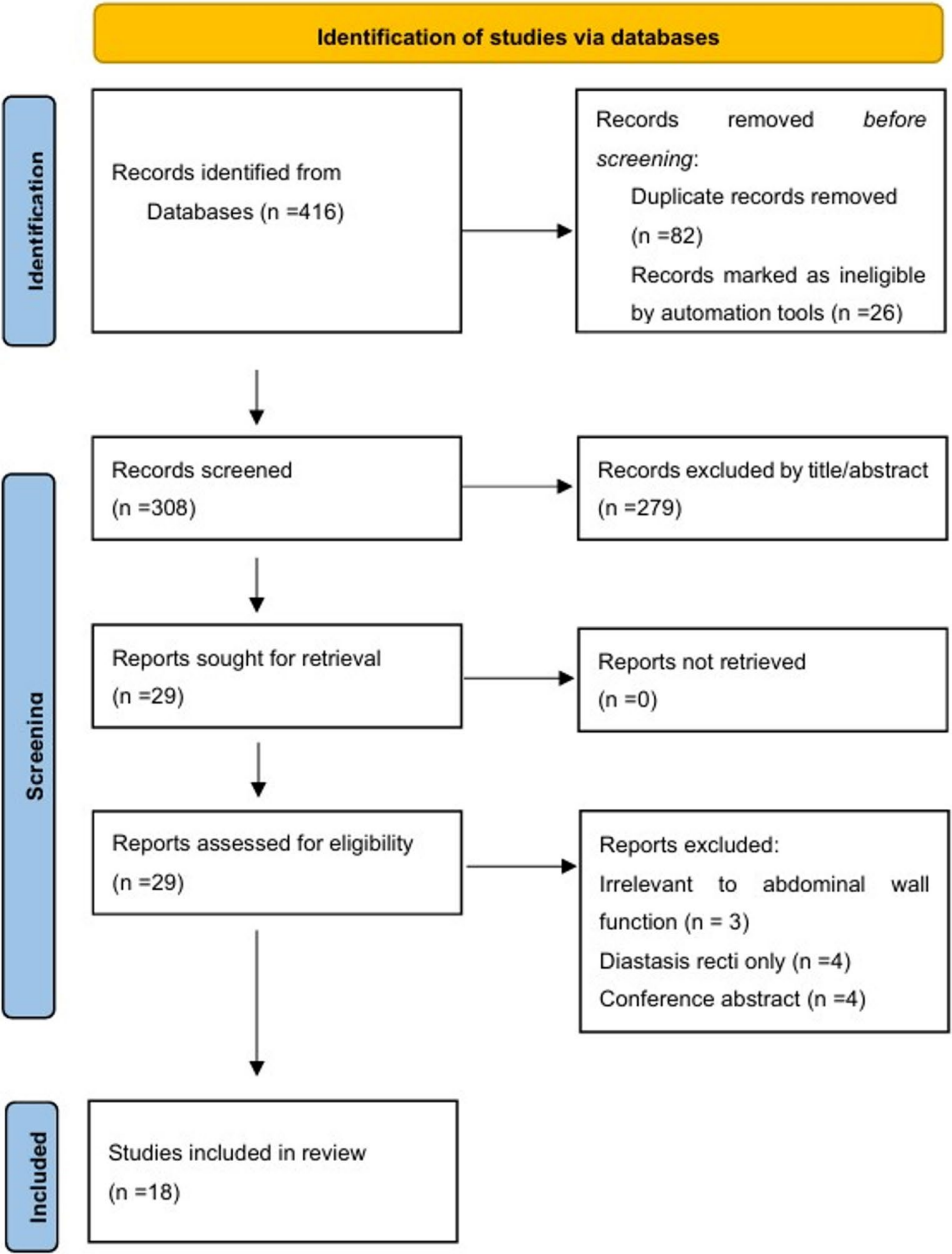
PRISMA diagram of search and screen process

The initial literature search yielded 416 results, of which 82 were duplicates and removed. Twenty-six studies were excluded as they were animal studies. The remaining 308 studies were screened by title and abstract, and 29 were retrieved. These 29 studies were reviewed in full text, and 18 studies were finally included [9–26]. The flowchart of the screening process in accordance with the PRISMA guidelines is shown in Fig. 1.

### Study characteristics

The studies included varied in their research designs and were categorized into observational studies and clinical trials based on the presence or absence of an intervention [21]. Among the observational studies, seven (38.9%) were cross-sectional, and two (11.1%) were cohort studies. Four (22.2%) of the clinical trials were randomized controlled trials (RCTs), and five (27.8%) were non-randomized trials.

### Population

The population comprised 892 participants, with a range of hernias: including ventral hernia (18.8%), incisional hernia (54.6%), and unilateral inguinal hernia (23.2%). In addition, thirty (3.4%) participants were healthy adults without a hernia. Their ages ranged from 12 to 76 years, with the overall sex distribution being 68.7% male and 31.3% female.

### Domains of function

**Fig. 2:**
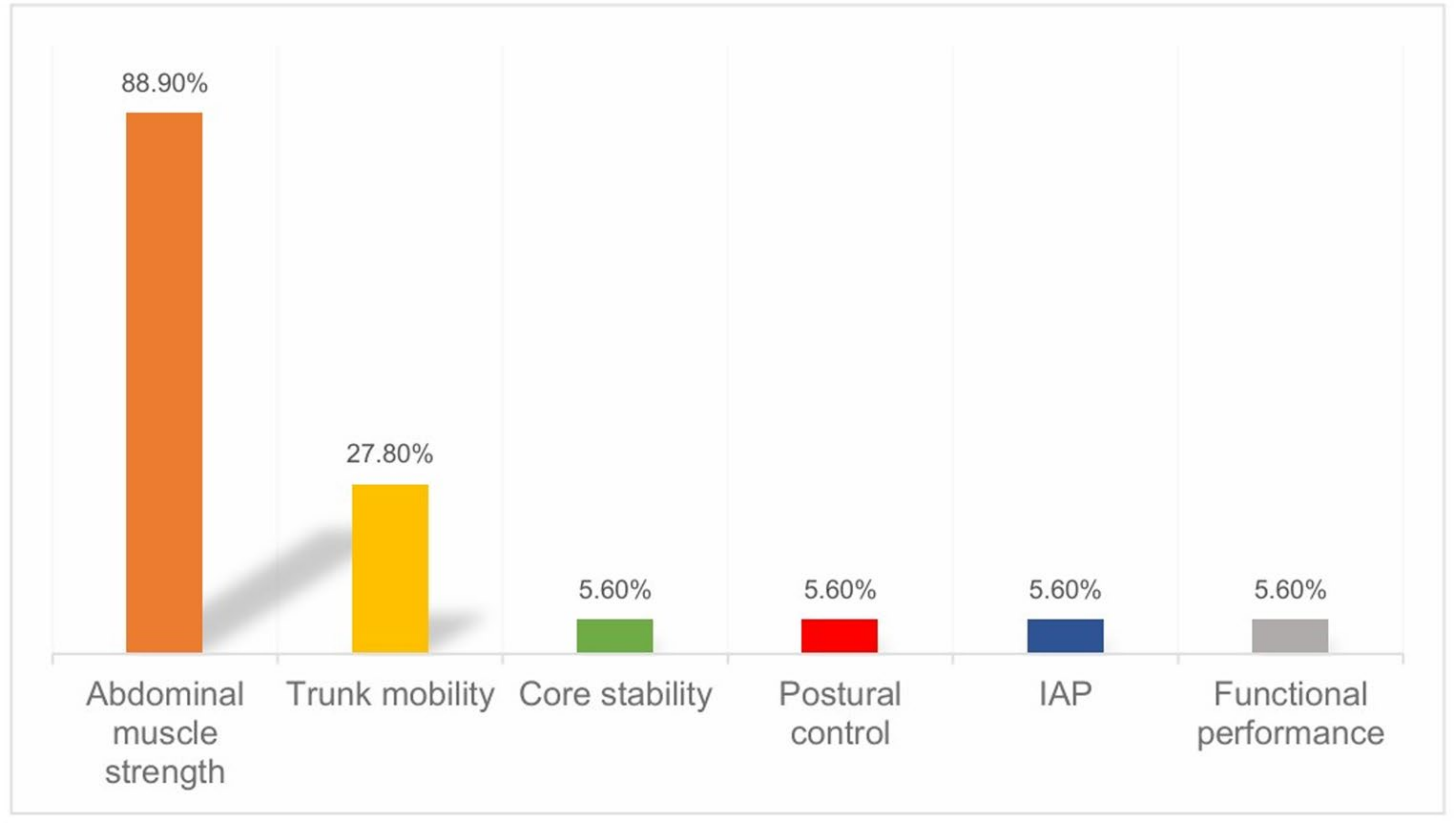
Distribution of function domains in AWF assessment

Different domains of AWF were identified in the included studies. They were classified into the following six main categories based on their focus and consensus from team: abdominal muscle strength, trunk mobility, core stability, postural control, IAP, and functional performance (assessed by 2-minute step test to reflect aerobic endurance). The categorization and frequency of AWF domains are shown in Fig. 2.

Abdominal muscle strength was assessed in 16 (88.9%) studies, which was the most frequently reported domain. Six studies evaluated isokinetic muscle strength, five measured isometric muscle strength, three on maximal voluntary isometric contraction (MVIC), and two on muscle endurance. Assessments were conducted using isokinetic or isometric dynamometers, surface electromyography (sEMG), and clinical tests.

Five (27.8%) studies assessed trunk flexion and extension by the isokinetic dynamometer, which reflected trunk mobility. However, it was not evaluated independently. Instead, the range of motion for trunk flexion and extension was evaluated and established for each participant prior to their isokinetic strength tests. In addition, the assessment of trunk mobility in these studies was limited to flexion and extension, trunk rotation was not assessed.

Although some studies have suggested an association between muscle strength and core stability, highlighting the important role of muscle strength in maintaining core stability, core stability was clearly defined and assessed in only one (5.6%) study. It refers to the ability to control the movements of the lumbo-pelvic-hip complex (LPHC) to ensure stability in functional activities. The core stability was assessed by the Sahrmann core stability and prone tests [16]. Postural control and balance were assessed in one (5.6%) study through barycenter variation evaluation, using a Nintendo Wii Balance Board (WBB) [13].

One (5.6%) study measured IAP, which was assessed by bladder technique, and was measured at end-expiration in the supine position, where the pressure recorded was expressed in mmHg [23].

Functional performance was assessed in one (5.6%) study, where a 2-minute step test was used to reflect aerobic endurance [20].

### Assessment methods and tools

Different assessment methods, including dynamometry, sEMG, and clinical tests, were used with various assessment tools to evaluate different domains of AWF, as summarized in Table 1.

**Table 1:** Summary of assessment methods and tools

Assessment method	Assessment tool/Clinical test	Function domains assessed		Reliability (✓, ×, unknown)	Validity (✓, ×, unknown)	
Dynamometry	Biodex isokinetic dynamometer [8, 10, 14, 20, 23, 24]		Muscle strength (Isokinetic and Isometric), trunk mobility	✓	✓	
	Good Strength Dynamometer [13]		Muscle strength (Isometric)	✓	×	
	Tension Dynamometer [9]		Muscle strength (Isometric)	Unknown	Unknown	
	Cybex isokinetic dynamometer [25]		Muscle strength (Isokinetic), trunk mobility	✓	✓	
Surface Electromyography	Neuropack MEB-9200 J/K EP/EMG Measuring System [17]		Muscle strength (MVIC)	Unknown	Unknown	
	BTS FREEEMG 1000 Surface Electromyography System [23]		Muscle strength (MVIC)	Unknown	×	
	NEUROSENS Surface Electromyography System [18]		Muscle strength (MVIC)	Unknown	✓	
Clinical tests	Trunk Raising test [22]		Muscle strength	Unknown	Unknown	
	Double Leg Lowering test [16, 22]		Muscle strength	Unknown	Unknown	
	Prone test [15]		Core stability	✓	Unknown	
	Sahrmann Core Stability test [15]		Core stability	✓	Unknown	
	Sit-up test [11]		Muscle endurance	Unknown	✓	
	2-minute step test [19]		Functional capacity	Unknown	Unknown	
Barycenter variation evaluation	Nintendo Wii Balance Board (WBB) [12]		Postural control/Balance	✓	✓	
Bladder pressure method	Foley catheter with pressure transducer [22]		IAP	✓	✓	

Six (33.3%) studies explored the application of the Biodex isokinetic dynamometer [9, 11, 15, 21, 24, 25]. Most of them reported measuring abdominal muscle strength, while one study specifically stated that it measured the strength of the rectus abdominis (RA) [19]. The Biodex isokinetic dynamometer evaluates the strength of the abdominal muscles in two dimensions. The isokinetic test is performed at different angular velocities with trunk flexion and extension movements. Parameters recorded include peak torque per body weight (PT/BW) and power. The isometric test assesses static contraction force at a fixed angle. All the tests were performed with participants adopting a standardized position, where they were seated with hips and knees at 90 degrees, the trunk and legs fixed with straps, and arms crossed over the chest to minimize compensation. In addition, the range of motion for trunk flexion and extension was assessed before muscle strength evaluation. All studies confirmed the validity and reliability of the Biodex isokinetic dynamometer, which objectively reflects abdominal muscle strength. The Good Strength dynamometer (Version 3.14 Bluetooth; Metitur Ltd, Finland), which is a portable isometric dynamometer, was used in one (5.6%) study. It measures isometric trunk flexor muscle strength, with participants adopting a seated position and resistance applied to their chest. This tool demonstrated high test-retest reliability, but the results showed no significant correlation with the International Physical Activity Questionnaire (IPAQ) [14]. The Tension dynamometer was used in one (5.6%) study, where its reliability and validity were not reported [10]. The Cybex isokinetic dynamometer (Cybex, Division of Lumex Inc., Ronkonkoma, NY, USA) was used in one (5.6%) study to measure isokinetic trunk flexor muscle strength, with PT/BW recorded as the parameter. This study suggested that the reliability and validity of this tool have been established in the literature [26].

Three sEMG tools were identified from the included studies, collecting MVIC signals to reflect muscle activation and strength. The Neuropack MEB-9200 J/K EP/EMG Measuring System was used in one (5.6%) study to capture MVIC data of the internal oblique (IO) and external oblique (EO) during trunk rotation [12]. The test position was developed from a standardized protocol. However, the reliability and validity of this protocol were not reported. Another study employed the BTS FREEEMG 1000 Surface Electromyography System to record MVIC signals of the RA, EO, and IO, but the reliability of this approach was not reported. This study found no significant correlation between sEMG signals and data from the Biodex isokinetic dynamometer, mainly because muscle activation level is not an independent determinant of torque generation during muscle contraction [18]. The NEUROSENS system was utilized in one study, recording MVIC signals from RA and EO. While the reliability of this tool was not reported, its construct validity was supported by the observed differences in sEMG amplitude between healthy individuals and patients with hernias [13].

The Trunk Raising (TR) and Double Leg Lowering (DLL) tests were utilized in two (11.1%) studies to evaluate abdominal muscle strength. Despite their common use in general clinical practice, the reliability and validity of these tests have not been established in hernia patients [17]. Moreover, one study found that the DLL test was not feasible for 75% of patients due to barriers such as obesity and pain [17]. The Sahrmann core stability and prone tests were employed in one (5.6%) study to assess core stability, demonstrating high test-retest reliability, but the validity was not reported [10]. The Sit-Up test (SUT) is a validated method of measuring abdominal muscle endurance and has been used in one (5.6%) study for this purpose [27]. Functional performance was evaluated using the 2-minute step test in one (5.6%) study, where the validity and reliability were not reported [20].

In addition to dynamometry, sEMG, and physical tests, barycenter variation evaluation was used to assess postural control and balance in one (5.6%) study. The Wii Balance Board (WBB; Nintendo, Kyoto, Japan) was applied as the assessment tool, which is suggested to be reliable and validated compared to the force plate [13]. The bladder pressure method, which is a standardized measurement, was used in only one (5.6%) study to assess IAP.

### Timepoints of assessment

Five (27.8%) studies performed only preoperative assessment [10, 17–19, 25]. One (5.6%) study recruited healthy people for a single session assessment [24]. Other studies (66.7%) performed both preoperative and postoperative assessments, with postoperative assessment timepoints varying from one week to one year postoperatively.

### Application of QoL assessment

In addition to AWF assessment, five (27.8%) studies reported assessments of QoL. Various QoL questionnaires were used, including the European Registry for Abdominal Wall Hernias Quality of Life (EuraHS-QoL), the Hernia-related Quality-of-Life Survey (HerQles), and the Short-Form 36 (SF-36). Among these studies, one discovered a significant improvement in both AWF and QoL after hernia surgery, suggesting a potential positive association between them [21]. This study further recommended using both validated subjective and objective measures to ensure a comprehensive assessment for hernia patients.

## Discussion

A previous systematic review which focused on abdominal muscle strength has described the equipment and tests employed for its assessment [5]. The present review aimed to identify and appraise all assessments of AWF (related to hernia assessment) reported in the literature, rather than limiting the scope solely to abdominal muscle strength. We intended to describe all the techniques that have been used to assess AWF, which could be applied to clinical practice. However, according to our search results, the majority (88.9%) of the included studies still focused on the assessment of muscle strength. Other aspects of AWF, such as trunk mobility, core stability, and postural control, were also reported, but less frequently. These findings reflected that muscle strength continues to be dominant in the assessment of AWF. This may be because this is the simplest to assess, however, it risks reducing a complex interaction of components of function to a single metric and perspective, which is not representative of in vivo function for patients.

Abdominal muscle function was evaluated objectively using dynamometry, sEMG, and clinical tests, with dynamometry being the most commonly used method. Four dynamometry tools were identified, where the Biodex isokinetic dynamometer was reported to be a reliable and validated tool for assessing isokinetic and isometric muscle strength [15, 21, 25]. However, its use was limited to assessing trunk flexors and extensors. In addition, the torque recorded during the assessment represented the combined output of bilateral trunk flexors. It does not provide side-to-side comparisons or detect muscle asymmetry. Beyond the expensive cost of the equipment, these are the key limitations of the use of the Biodex isokinetic dynamometer. Nevertheless, it remains the most commonly used and widely recognized tool for assessing AWF of hernia patients in research. This was further reflected by a study using the Good Strength dynamometer, which intended to validate it against the Biodex isokinetic dynamometer, although there was no gold standard in the assessment of abdominal muscle function [14]. Another advantage of the Biodex isokinetic dynamometer is its ability to assess the range of motion for trunk flexion and extension. However, trunk rotation has not been reported to date.

The sEMG could address some limitations of the Biodex isokinetic dynamometer. It receives signals from the attached electrodes and can, therefore, reflect the activation of targeted superficial abdominal muscles. In addition, sEMG can be employed to evaluate muscle activation during trunk rotation, including the activation of EO and IO. However, current literature reflects notable variability in the selection of muscles assessed, leading to inconsistency across studies. A key limitation of sEMG is its inability to detect deep muscle activation (e.g., transversus abdominis). Compared to the Biodex isokinetic dynamometer, the application of sEMG in hernia patients remains limited, and its validity has not been widely established [18].

Additionally, various physical tests were identified for the assessment of abdominal muscle function. These tests can be performed without specific equipment, making them convenient to use in practice. However, their validity and reliability have not been well established in the literature. In addition, the results of physical tests could be influenced by the examiner’s experience and subjective interpretation, leading to potential bias and reduced accuracy. One study further indicated that the DLL test was not feasible for 75% of the participants, due to barriers like pain and obesity [17]. Although some physical tests, such as the prone and the Sahrmann Core Stability tests, demonstrate high reliability in assessing core stability, they were not widely used in hernia patients. Only one study reported their use [16]. To apply these physical tests in clinical practice, further research is required to investigate their use in hernia patients, especially regarding their feasibility, reliability, and validity.

Although muscle strength assessments remain the dominant method of assessing AWF, each assessment method has its limitations. The Biodex dynamometer is costly and space-demanding, sEMG provides information on superficial muscle activation but is technically limited, where clinical tests are simple and accessible but lack validation and standardization. There is also a lack of high-quality, low-risk-of-bias RCTs to support their use in hernia patients. In addition, there is considerable inconsistency across different assessment methods, and the timepoints of evaluations vary significantly across studies. These limitations result in an absence of clear guidance in clinical practice, creating barriers to selecting appropriate measurements of AWF. Furthermore, existing studies generally fail to integrate objective assessments of AWF with subjective evaluations of PROMS in a comprehensive manner, where they provide different information for the assessment of patients’ function. Objective assessments can provide quantitative data on AWF, but they do not directly capture the patients’ functional experience. In contrast, PROMS, such as pain and QoL, reflect the perceived experience of impaired AWF. PROMS are therefore important components of functional recovery and patient satisfaction, and we suggest that future research combine objective assessments and PROMS to reflect AWF more comprehensively.

Overall, this review shows that AWF is an overlooked and under-assessed component of how hernias affect patients, and what effect interventions have. This is in contrast to other accepted components of PROMS, for example, pain, activity and mood which have multiple validated assessment tools. Some of this may be explained by the relative difficulty in quantifying AWF using laboratory or other specialist equipment, but perhaps also by a lack of awareness of consensus on what constitutes AWF. Although this scoping review identified key domains of abdominal wall function, including muscle strength, core stability, trunk mobility, postural control, IAP, and functional performance, it has not been universally accepted. We suggest that the community develop a consensus on how AWF should be considered and defined, and also consider how we can assess this in a reproducible and consistent way.

This scoping review has some limitations. Due to the heterogeneity of research designs among the included studies, we did not perform a risk of bias assessment. However, this does not affect our findings, as our objective was to explore all available assessment methods rather than to evaluate study quality.

## Conclusion

This scoping review discovered significant differences in assessments of AWF reported in the literature. The evaluation of abdominal muscle strength remains dominant, but there is inconsistency regarding the tools and tests used and the timepoints of evaluation. In addition, research combining objective and subjective assessments remains limited. Currently, no assessment method can provide a comprehensive reflection of AWF. Therefore, future research is required to develop or modify existing assessment methods to fully reflect AWF, which can then be incorporated into clinical and research practice.
